# Developing a process for assessing the safety of a digital mental health intervention and gaining regulatory approval: a case study and academic’s guide

**DOI:** 10.1186/s13063-024-08421-1

**Published:** 2024-09-10

**Authors:** Rayan Taher, Charlotte L. Hall, Aislinn D Gomez Bergin, Neha Gupta, Clare Heaysman, Pamela Jacobsen, Thomas Kabir, Nayan Kalnad, Jeroen Keppens, Che-Wei Hsu, Philip McGuire, Emmanuelle Peters, Sukhi Shergill, Daniel Stahl, Ben Wensley Stock, Jenny Yiend

**Affiliations:** 1 Department of Psychosis Studies, Institute of Psychiatry, Psychology & Neuroscience, King’s College London, London, UK; 2 Institute of Mental Health, School of Medicine, National Institute for Health and Care Research MindTech MedTech Co-Operative, University of Nottingham, Nottingham, UK; 3 School of Computer Science, University of Nottingham, Nottingham, UK; 4 Avegen Health, London, UK; 5 London Institute for Healthcare Engineering, King’s College London, London, UK; 6 Department of Psychology, Addiction and Mental Health Group, Bath Centre for Mindfulness and Compassion, University of Bath, Bath, UK; 7 McPin Foundation, London, UK; 8 Department of Informatics, King’s College London, London, UK; 9 Department of Psychological Medicine, Dunedin School of Medicine, University of Otago, Otago, New Zealand; 10 Department of Psychiatry, University of Oxford, Oxford, UK; 11 Department of Psychology, Institute of Psychiatry, Psychology and Neuroscience, King’s College London, London, UK; 12 Kent and Medway Medical School, Canterbury, Kent UK; 13 Department of Biostatistics and Health Informatics, Institute of Psychiatry, Psychology & Neuroscience, King’s College London, London, UK; 14 Wensley Stock Ltd, Wiltshire, UK

**Keywords:** Safety, Regulatory approval, MHRA, Digital mental health, Digital mental health interventions, Adverse events, Medical device, Software as a medical device, SaMD, Notification of No Objection

## Abstract

**Background:**

The field of digital mental health has followed an exponential growth trajectory in recent years. While the evidence base has increased significantly, its adoption within health and care services has been slowed by several challenges, including a lack of knowledge from researchers regarding how to navigate the pathway for mandatory regulatory approval. This paper details the steps that a team must take to achieve the required approvals to carry out a research study using a novel digital mental health intervention. We used a randomised controlled trial of a digital mental health intervention called STOP (Successful Treatment of Paranoia) as a worked example.

**Methods:**

The methods section explains the two main objectives that are required to achieve regulatory approval (MHRA Notification of No Objection) and the detailed steps involved within each, as carried out for the STOP trial. First, the existing safety of digital mental health interventions must be demonstrated. This can refer to literature reviews, any feasibility/pilot safety data, and requires a risk management plan. Second, a detailed plan to further evaluate the safety of the digital mental health intervention is needed. As part of this we describe the STOP study’s development of a framework for categorising adverse events and based on this framework, a tool to collect adverse event data.

**Results:**

We present literature review results, safety-related feasibility study findings and the full risk management plan for STOP, which addressed 26 possible hazards, and included the 6-point scales developed to quantify the probability and severity of typical risks involved when a psychiatric population receives a digital intervention without the direct support of a therapist. We also present an Adverse Event Category Framework for Digital Therapeutic Devices and the Adverse Events Checklist—which assesses 15 different categories of adverse events—that was constructed from this and used in the STOP trial.

**Conclusions:**

The example shared in this paper serves as a guide for academics and professionals working in the field of digital mental health. It provides insights into the safety assessment requirements of regulatory bodies when a clinical investigation of a digital mental health intervention is proposed. Methods, scales and tools that could easily be adapted for use in other similar research are presented, with the expectation that these will assist other researchers in the field seeking regulatory approval for digital mental health products.

**Supplementary Information:**

The online version contains supplementary material available at 10.1186/s13063-024-08421-1.

## Background

The field of digital mental health interventions (DMHIs) has followed an exponential growth trajectory in recent years [1]. DMHIs typically involve mental health interventions, such as cognitive behavioural therapy, delivered via digital technologies, such as smartphones, and can either be completed as self-directed interventions or blended alongside synchronous (e.g., face-to-face or videoconference) or asynchronous (e.g., email or text message) clinical support [2]. The main benefit of these interventions is delivering evidence-based care to a large number of people with limited clinical resources [3]. While the evidence base has increased significantly, the adoption of these interventions within health and care services has been slowed by several challenges, including a lack of knowledge from researchers regarding how to navigate the pathway for mandatory regulatory approval. In the UK, DMHIs must meet the standard of evidence set by the National Institute of Health and Care Excellence (NICE) for adoption within the National Health Service (NHS) [4]. For DMHIs that are developed to diagnose, prevent, monitor, treat, or alleviate a mental health condition, this may include regulation as a “Software as a Medical Device” (SaMD) by the Medicines and Healthcare products Regulatory Agency (MHRA) [5]. The regulatory process ensures that devices used within the health and social care context are safe and effective.

In some cases, research will involve digital therapeutics that are already in use and carry a CE or UKCA mark. In this case, the therapeutic’s safety and effectiveness has already been established (and is maintained either through self-certification by the manufacturer or, for higher risk devices, through the use of a “Notified Body”: a government-approved organisation that ensures the device continues to conform to the required standards). However, early-stage digital therapeutics will not yet bear a CE/UKCA mark and are therefore required to obtain a specific form of regulatory approval from the MHRA (called “Notification of No Objection”; NoNO) before being used in research, in addition to the usual ethical approvals [6]. The NoNO regulatory process requires that safety and effectiveness data collection are the primary purpose of a clinical investigation, with the overall aim being to establish whether the benefits of the device outweigh its risks. This places a number of constraints and requirements upon how researchers design their investigations and write their protocols, the most obvious being that rigorous safety assessment is paramount. The present paper is intended to help academics who are interested in digital therapeutics, but unfamiliar with medical device safety assessment, to navigate a course through this complex regulatory field.

Although the research proposal for which NoNO is sought will, as already explained, need to have safety as a primary outcome, obtaining NoNO also requires the research team to demonstrate the safety of their device before the proposed investigation can be approved [6]. To understand this apparent contradiction, it is crucial to appreciate that safety assessment is considered an inherently iterative process: preliminary safety data must be presented in order to justify collecting more detailed safety data. This can be done by providing a summary of the existing device safety information using all possible sources (e.g. prototypes, user testing, pilot or feasibility data, qualitative information); a risk management plan (identifying all possible hazards, their potential impact and mitigations) and a detailed plan for safety assessment in the proposed clinical investigation (such as collecting and assessing any untoward medical occurrences [7], usually called adverse events (AEs)) [6].

However, researchers investigating DMHIs face specific challenges when proposing a safety assessment plan. Notably, MHRA guidance was developed in consideration of medical devices used in clinical contexts such as surgery and pharmacological interventions and was not designed to accommodate the unique safety considerations relevant to DMHIs. Additionally, the guidelines used in research for assessing the safety of DMHIs are borrowed from the medical and pharmaceutical fields, such as the “International Council for Harmonisation of Technical Requirements for Pharmaceuticals for Human Use—Good Clinical Practice” (ICH-GCP) guidelines [8]. These medical guidelines do not transfer well to assessing the safety of both digital and non-digital psychological interventions because of the fundamental differences between pharmacological and psychological approaches to treatment [8]. For example, biological responses to medicines usually occur rapidly and can be objectively measured, whereas psychological responses to therapy rely heavily on patients’ self-reported symptoms, can be hard to disentangle from other contextual factors, and intervention effects can take days, weeks or even months to emerge. As others have also identified, using medical definitions and processes to assess the safety of non-medical interventions such as DMHIs and behaviour change interventions can be unhelpful. It can overcomplicate the process of safety assessment, and lead to missing important harms [8–10].

These concerns have already been raised in trials assessing the safety of behaviour change interventions [8]. For example, in a qualitative study on recording harms in RCTs for behaviour change interventions, experts emphasised the need for harm recording to be proportionate and focused on harms that are plausibly linked (i.e. related) to the intervention under study [34]. It is likely that medical processes are being used to assess the safety of DMHIs, because there are no regulatory or standard safety assessment processes in place for face-to-face mental health interventions [9]. This is surprising given that most adverse events/side effects are common to both face-to-face mental health interventions and DMHIs (e.g. short-term deterioration, novel symptoms, and non-response) [10–12]. The one area that differs is, of course, technical and device-related harms.

Two recent reviews found that the identification and categorisation of AEs in DMHI trials was inconsistent and often inadequate [3, 11]. This was similar to findings of a review on safety assessment in non-pharmacological psychological, behavioural and lifestyle interventions [8]. It is essential that harmonised standards tailored specifically to the needs of DMHIs are developed. Support mechanisms can then be implemented to assist manufacturers and researchers to understand and adhere to these guidelines*.* In the absence of these, the purpose of this paper is to share a worked example of how our clinical trial team successfully applied and received the MHRA’s NoNO for STOP (Successful Treatment for Paranoia).

STOP is a mobile app DMHI that uses Cognitive Bias Modification for paranoia (CBM-pa) to reduce symptoms of paranoia [12, 13]. STOP consists of 12 weekly sessions of about 40 min each. In each session, the user is presented with 40 ambiguous scenarios that could be interpreted in a paranoid manner. Users are then guided to reevaluate each scenario in a non-paranoid way by completing words and answering questions designed to suggest alternative meanings. The goal is to gradually retrain the brain to assume non-paranoid meanings of ambiguous situations that occur in daily life, which has been shown to reduce paranoid symptoms. More information about STOP and its development is provided elsewhere [13]. Using STOP as our example, we aimed to provide valuable insights to other research teams undertaking clinical investigations of DMHIs, particularly those requiring regulatory (e.g., MHRA) approval and guidance in the assessment of safety in DMHI research.

## Methods

### Participants

The work presented in this paper was collaboratively completed by academics and clinicians in the field of digital mental health, an expert regulatory consultant, device manufacturers (Avegen), a clinical trials unit at a university, and representatives from an organisation working with experts by experience (the McPin Foundation). Participants varied at each stage; more detail is provided below per task. See Appendix A for the full list of participants.

### Procedure

To assess the safety of STOP (ISRCTN17754650) and obtain the MHRA’s NoNO the STOP team needed to achieve two main objectives:

A. Demonstrate existing safety.
B. Evaluate safety within the proposed research for which approval was being sought.

#### Demonstrating existing safety

This objective was achieved by completing three separate tasks; an empirical feasibility study; relevant literature searches and the creation of a comprehensive Risk Management Plan.

#### Feasibility study

The research team needed to present current safety data relevant to STOP such as previous publications, feasibility or pilot studies from the same or similar devices/ interventions. To achieve this, the team referred to a previously conducted study that had assessed the intervention’s feasibility and safety as a desktop intervention [12]. The feasibility study included two arms: treatment (CBM-pa which is a 6-session version of the therapeutic intervention used in STOP but delivered using a desktop computer) and an active control (a version of the same 6 session desktop programme with the same design and format as CBM-pa, except the content was neutral and should not trigger paranoid thoughts) [12]. CBM-pa works in the same way as STOP (see “Background”) by presenting users with a scenario that could be interpreted in a paranoid way and then, using word tasks and questions, helping participants to interpret the scenario in a nonparanoid, benign way [12]. Sixty-three outpatients with clinically significant paranoia participated in the feasibility study and were randomised to either the treatment or control group [12].

The feasibility study assessed safety by measuring whether presenting participants with these potentially paranoia-inducing scenarios was distressing or provoking for them, using visual analogue scales (VAS) completed before and after every session and measuring state anxiety, sadness, paranoia and friendliness. These data were used as proxy safety data relevant to STOP because STOP uses the same content and procedures as CBM-pa but delivered in a different format (mobile app vs. desktop) and the sample size of the feasibility studies are usually small [12, 13].

#### Literature searches and regulatory databases

Second, three members of the research team (RT, CH, JY) conducted two literature searches to identity any published safety data that might be relevant to STOP or any equivalent intervention. These literature reviews are different to those used in academia and thus follow a different structure [14]. The team worked with an independent regulatory consultant on these to make sure we followed industry and regulatory standards. See Appendix B for more details around the methodology used in these two separate literature reviews. As part of this review, FDA databases were also searched for similar devices and any reported adverse events.

#### Risk management plan

The risk management plan was created collaboratively by key members of the STOP trial academic team and other project stakeholders, including members from a Lived Experience Advisory Panel, members of the software manufacturer and a regulatory consultant (see Appendix A, column 1, for full details).

Under this step, and by using the knowledge that arose from the feasibility study and the review, we developed a risk management plan. This step is required to demonstrate to regulators that the research team has listed all possible hazards, documented what harms might result from each hazard and identified actions or changes that will mitigate every risk entry as far as possible. Regulators require a comprehensive risk analysis specific to the product, showing a clear understanding of the following stepwise process: hazard, harm, initial risk rating, risk controls/ mitigations, revised risk ratings, identification of residual risks and final demonstration that the expected product benefits outweigh the identified residual risks, which should have been reduced as far as possible. In STOP’s case, a residual risk matrix (likelihood × severity) demonstrated that there were no residual medium or high risks.

The risk management plan described above was implemented by carrying out the following activities:

1. Hazard identification

To develop a risk management plan, the team first needed to develop a list of all potential hazards that participants taking part in the trial could be exposed to and articulate the harms that could result. Based on ISO 14971, which is a standard for risk management for medical devices, hazards are defined as “a potential source of harm” and harms are defined as “injury or damage to the health of people, or damage to property or the environment” [15]. This was done during a 1-h consensus meeting with an expert regulatory consultant, two representatives from the manufacturer, two clinicians, two academics, and one representative of a user organisation. During the consensus meeting all participants brainstormed possible hazards and articulated, through discussion, the harm it could lead to. The meeting resulted in a comprehensive spreadsheet of hazards and corresponding harms. The spreadsheet was compiled and circulated for members to review, revise and populate with any further suggestions.

2. Hazard analysis

After identifying hazards, a hazard analysis needs to be performed. While manufacturers may be experienced in providing this for the technical side of their products, the majority of hazards for DMHIs will be related to clinical risks. Most manufacturers will be unable to assess these and will require the clinical and academic team to become conversant with applying and interpreting risk assessment procedures. To this end, the STOP clinical and academic team received training in risk assessment from a regulatory consultant who worked with them to implement the process outlined below.

The first step in a hazard analysis is to quantify the probability (likelihood) and severity (impact) of each identified hazard. First, one must assess the probability that each hazard will lead to the specified harm. One must assume that the hazard has occurred and then ask oneself “how likely is the harm to now happen?”. Some of these may be fairly standard assessments or known within the digital industry, for example, should a participant stare at the screen for longer than advised, how likely is it that they will experience physical side effects such as eyestrain, fatigue or headache? However, in many cases nuanced clinical judgements are required to make this assessment. For example, how likely is it that the participant’s condition will worsen in the short term as a result of engaging with the content of the therapy? Second, one must assess the severity and impact, should that harm occur. For example, were eye strain, fatigue or headache to occur as a result of using the device how severe could those effects be at their worst? Severity in risk assessment can be operationalised in these terms:

i. The duration of harmful effects.
ii. The level of intervention or support needed in response to the effects.
iii. The possibility and extent of any long-lasting or permanent impact.

Central to the STOP hazard analysis was the need to create bespoke probability and severity scales relevant to the clinical therapeutic context. These were carefully devised by consensus discussion between the regulatory expert consultant and members of the clinical academic team to agree the most appropriate exact thresholds and wording for both dimensions. The application of the preliminary hazard analysis for STOP was a quantitative assessment of each individual risk entry (i.e., hazard and corresponding harm) against the criteria for probability and severity outlined above. The product of these two scores yields a “risk score”. It is important to note that these risk scores were based on expert consensus estimations derived from their knowledge of the literature and field experience. In line with standard practice in the field of risk analysis, no formal validation was conducted.

3. Risk control and re-evaluation

In common with all risk assessments, the next stage was to work through each of the identified risks outlining all the “risk control” actions (i.e., mitigations) that could be taken to reduce the identified risk to participants as far as possible. Each risk is then re-evaluated in terms of its probability and severity yielding a revised (“post risk control”) risk score. Finally, a risk acceptability management plan is implemented where various actions are specified for any residual risks that cannot be reduced any further, for example adding “warnings” and “cautions” to device details and labelling. These serve to alert the user to important residual risks that cannot be addressed in any other way. One residual risk that is common to mental health interventions is the possibility of users being distressed when presented with information that relates to the mental health condition they are living with.

In the case of STOP, residual risks were managed using a variety of processes, depending on the nature of the risk. This included, for example, warnings (e.g. “If negative feelings or symptoms worsen as result of using this app for more than a day, please contact your support team and cease use of the STOP app until advised further”), fortnightly check-in phone calls with researchers; a dedicated, in-app 24-h study helpline number and use of an inbuilt mood-tracking algorithm to trigger researcher alerts. Further details of these are provided below.

#### Evaluating the safety of a DMHI within the proposed research study

After demonstrating the current safety of STOP as seen in section A of this paper, the team needed to demonstrate how the safety of STOP would be assessed in the proposed clinical trial. Any assessment of safety will involve collecting data about the occurrence of adverse events, both related and unrelated, to the trial. For STOP, we planned to do this proactively and regularly in line with recent recommendations [11]. We therefore needed an overarching framework to organise and classify the large quantity of adverse event information that was likely given the larger sample size (273) and length of time each participant would spend in the trial (6 months). We therefore devised an adverse event classification framework as follows.

#### AE classification framework development

1. Literature review

First a brief narrative literature review (conducted within the limited, 60-day time window of the regulatory approval pathway) was carried out to identify any publications in the last 10 years [03/23/2012–03/23/2022] that discussed how adverse events were assessed, coded or categorised in psychiatric populations receiving psychological interventions (digital or non-digital). We combined the categories and definitions identified in the outputs of the literature review to create a first working draft of a classification framework.

2. Expert consultation

We then carried out an expert consultation involving key members of the STOP trial academic team and other project stakeholders. This included the McPin Foundation, key members of the software manufacturer, a regulatory consultant and key external members of the trial committees. Full details are given in Appendix A. The classification framework working draft was shared with this group to review and comment upon. The group was invited to edit, remove or add categories or examples. Where any conflicts or differences of opinion emerged, these were resolved by group discussion and consensus using virtual meeting and/or email communications. This resulted in a finalised ‘Adverse Event Category Framework for Digital Therapeutic Devices’ which is provided in the “Results” section.

#### Proposed safety plan for the trial

To appropriately and sufficiently assess the safety of a DMHI, regulators expect to see safety positioned as the primary outcome in the proposed study, alongside efficacy. In the STOP trial, this was done by adjustment of the protocol in three ways.

First, we built-in proactive, fortnightly collection of AE data for each participant throughout the entire trial (including throughout the follow-up period) in both arms, using a custom designed checklist based upon the Adverse Events Category Framework for digital devices described above. Even though collecting AE data in both arms is resource-intensive, it is important, as shown in a recent systematic review [11]. These data enable researchers to statistically compare the prevalence of AEs in the treatment and control arms, allowing for conclusions about the safety of the DMHI. The checklist was developed from the framework, customised to the STOP trial and designed to be administered by researchers during a 10-min phone or video interview with participants. Customisation included adding introductory scripting, one or more prompt questions under each adverse event category, examples of typical events for researchers’ reference and reordering/ grouping categories and questions to optimise efficiency and acceptability of the delivery. According to the ICH-GCP guidelines, all AE data need to be categorised based on seriousness, severity, relatedness and expectedness [16]. This was done following standard guidance widely available across clinical trials units (see Appendix C for further details). The checklist was devised to incorporate the first three of these evaluations (seriousness, severity, relatedness). By definition, any event that fell within one of the listed Adverse Event Categories was considered “expected” (i.e. anticipated). Items that had not been foreseen and were therefore classed as “unexpected” were listed under the “Other” category heading. The resulting Adverse Events Checklist for the STOP trial is presented in the “Results” section.

In response to regulatory safety concerns, the frequency of AE data collection calls was increased to once a week for any participants identified as high risk. High-risk participants were identified at baseline using a cut-off score on a Persuadability/Suggestibility scale [17] (higher suggestibility can lead to higher risk, as the intervention aims to foster nonparanoid and trusting thoughts) and a suicide risk assessment, and throughout the trial using a suicide assessment that was administered on a weekly/biweekly basis. In addition, researchers recorded any AE that was spontaneously reported by the participant at any other contact. Note that it is crucial to collect AE data using identical methods for both the intervention and control groups even if the trial is unblinded as the control group serves as an important baseline for adverse event occurrences.

Second, we built in safety monitoring within the device. An algorithm was used to trigger an alert to researchers whenever a participant had a worsening of state mood on self-reported levels of paranoia, anxiety or sadness across a weekly treatment session (using visual analogue scale in-app pre/post session assessments; see Supplementary File 1) on 3 consecutive occasions. Researchers would then make a follow-up call to check in on the participant, collect further information and safety data and decide if follow-up action was needed (for example alerting a GP or clinical care team).

Third, we added a specific outcome measure relevant to safety, namely the Negative Effects Questionnaire (NEQ) administered once at the end of the intervention (end of treatment). The NEQ is a 20-item self-report measure [18]. It was developed using the results from Rozental et al., (2014)’s consensus statement on the negative effects of internet interventions [18], and studies aimed at investigating the negative effects of psychotherapy [18, 19]. It is used to collect data on the negative effects experienced by patients/users during treatment, their severity and whether they were related to the intervention or other circumstances [19]. The NEQ is a reliable and valid measure with an internal consistency of *α* = 0.95 [19].

## Results

### Demonstrating the existing safety of the DMHI

#### Feasibility study

The feasibility study main outcome paper reported no adverse events or serious adverse events and an “absence of evidence of any harmful effects on state mood and the practicality of the protocol as delivered” [12] The results from the VAS showed that there was no evidence of significant short-term detrimental effects on anxiety, sadness, paranoia or friendliness in the intervention group compared to the control group, suggesting that the intervention did not exacerbate negative mood, or pose any risk of harm to patients with distressing paranoia [12]. These data are provided in Supplementary File 1. The STOP study team used these combined findings to argue in support of the safety of STOP based on its similarity in therapeutic content to CBM-pa.

#### Literature search and regulatory databases

1. Results of literature review 1 (Device use or experience):

The search for the first literature review resulted in 14 included studies. See Appendix D for the respective PRISMA flowchart. Results showed evidence that cognitive impairment in this population does not affect engagement with digital interventions [20]. There was evidence suggesting that digital interventions are effective at improving social functioning [21], memory [22], educational and vocational attainment [23], personal recovery [24], and alleviating loneliness [25] in psychotic disorders. Some digital interventions used in this population aimed to monitor symptoms such as sleep [26], and psychotic symptoms [24]. The results of a previous literature search [27] showed that there were three digital mental health interventions that have been developed to improve symptoms in individuals struggling with psychosis [21, 23, 25]. The review included eight papers on smartphone-based interventions for psychosis, of which three were protocols, two were feasibility studies, two were pilot RCTs and only one was an RCT with a sample of 36 participants. This RCT found that participants who used Actissit (a Cognitive Behavioural Therapy based app for psychosis) plus treatment as usual experienced better improvements psychotic symptoms compared to those who used a symptom monitoring app plus treatment as usual [28].

The data on the *use or experience* of digital therapies to monitor, reduce symptoms or improve recovery in this population were promising but still limited. Larger randomised controlled trials are needed. There was no study on the use or experience of digital mental health interventions in a sample specifically defined by paranoid symptomatology except for the feasibility study precursor to the STOP [11]. For that, the literature search criteria was expanded to include devices that address psychosis in general to find comparable studies.

2. Results of literature review 2 (Device safety):

The search for the second literature review resulted in five included studies. See Appendix D for the respective PRISMA flowchart. Although the literature on the safety of digital mental health interventions targeting paranoia/psychosis is limited, all the current studies demonstrated positive safety outcomes [21, 23]. A number of studies assessed the safety of the Horyzons—an online social media-based intervention that was designed to enhance social functioning in individuals with a first episode of psychosis; the studies found Horyzons safe to use (no incidents) and Horyzons users reported feeling safe and empowered [23, 29, 30]. A social media-based intervention called (MOMENTUM), which aims to improve social functioning in “at high-risk mental state” young individuals, was found to be safe to use [29, 30]. A randomised controlled trial (*N* = 36) of Actissit—a CBT-informed mobile phone app for people who have experienced psychosis—found it safe to use (no serious adverse events) [28]. Finally, a randomised clinical trial (*N* = 41) assessing the EMPOWER app (Early signs Monitoring to Prevent relapse in psychosis and prOmote Wellbeing, Engagement and Recovery) reported 9 adverse events that were related to the app such as increased feelings of paranoia, increased fear of relapse and technical issues [31]. Findings were in line with those of a systematic review on the digital interventions for early psychosis where all eight smartphone-based interventions under study were found to be safe [27].

The clinical data appraisal tools for both literature reviews are provided in Appendix C.

3. Regulatory databases

There were no safety concerns raised from the review of the regulatory databases.

#### Risk management plan

1. Hazard identification

In total 26 unique hazards and their corresponding harms were identified, which are listed in full in Appendix E.

2. Hazard analysis

The team defined likelihood/probability and severity for the proposed study as explained in the “Methods” section. Table 1 shows the final operationalised definition of probability and Table 2 shows the equivalent for severity.

**Table 1:** The operationalised definition of probability for the hazards posed by STOP

Likelihood/Probability		Proportion of patients
1	Improbable	0–5% patients
2	Remote	5–10% patients
3	Occasional	10–25% patients
4	Potential	25–50% patients
5	Probable	> 50% patients
6	Frequent	> 70% patients

**Table 2:** The operationalised definition of severity for the hazards posed by STOP

#	Severity	Impact
1	Minor level of harm Negligible	No effects or transient effects (minutes to hours). Participant is practised in dealing with similar effects without needing additional support. No lasting physical or psychological harm
2	Minor level of harm Minor	Short lived effects (up to 1 week). Participant is practised in dealing with similar effects without needing additional support. No lasting physical or psychological harm
3	Moderate level of harm - Moderate	Effects sustained for at least 1 week but that participant is used to dealing with either alone or with existing support already in place. No lasting physical or psychological harm
4	Moderate level of harm- Significant	Medium term effects sustained for at least **4** weeks that are clinically significant and **may require additional clinical support** beyond that currently received (e.g. contact with clinical services for assessment and possible treatment). Effects can be **fully mitigated** by additional support or short-term clinical intervention without lasting physical or psychological harm
5	Significant level of harm Catastrophic	Significant effects sustained for at least **6** months that are clinically significant and **require intervention** from clinical services for management and treatment. May be some **lasting effects that cannot be eliminated** (e.g. traumatic memory/ re-experiencing of a damaging event)
6	Significant level of harm Critical	Significant effects requiring **emergency clinical intervention** and treatment and leading **to long-term serious harm or death**

Afterwards, the team used these definitions to rate the probability and severity of every identified hazard. Probability refers to *the likelihood that the identified hazardous situation will lead to the specified harm*. A risk score was calculated for each hazard entry by multiplying the probability and severity scores.

3. Risk control and re-evaluation

Under this step, the study team identified all measures they could take to reduce each risk entry as much as possible, listing these as “risk controls”. They then recalculated new probability, severity and risk scores under the assumption that the stated risk controls were effective. Before implementing risk-control strategies, the highest risk score was 20 out of 36. After applying these strategies, the highest risk score was reduced to 9 out of 36. These quantitative ratings and products are shown in Appendix F, which constitutes the final STOP Hazard Analysis, and was a key requirement of the submission for regulatory approval. One new insight that emerged from the consultation was the need for a product recall feature that could operate at either an individual or entire cohort level. This requirement was a crucial safety attribute, for use in the unlikely event that access to the app had to be immediately terminated. At the individual level access could be revoked by account deactivation. At the cohort level, the technical team implemented a “recall switch” feature in the app to enable the recall, and a corresponding participant facing message.^1^

### Evaluating the safety of a DMHI within the proposed research study

#### AE classification framework development

The literature review and the expert consultation that we conducted as described in the “Methods” section resulted in a finalised “Adverse Event Category Framework for Digital Therapeutic Devices” (See Table 3) . This framework was informed by three key publications arising from the literature review that discussed a range of classes of negative effects in psychotherapy [18, 32, 33]. Our framework was then used to identify the “anticipated” AEs for the STOP trial. As such, it might be applicable to all DMHIs that are in the form of a mobile app. The AEs collected are not exclusive to STOP but might not all be relevant or be comprehensive of all possible AEs for another DMHI. Professionals testing a DMHI delivered in a different format (virtual reality for example) and/or targeting a different population would need to make suitable adjustments and might even wish to incorporate additional AE categories specific to their device’s safety profile. However, this framework is recommended as a potentially useful starting point for any DMHI.

**Table 3:** Adverse event category framework for digital therapeutic devices

AE category	AE category description
Symptom exacerbation/deterioration	Any increase in clinical symptoms or deterioration in mental health condition, for example due to: • natural worsening of a patient’s clinical condition • some aspect of the study or device (e.g. therapeutic content which is triggering; device use creating anxiety)
Novel symptoms	Occurrence of novel symptoms (e.g. panic attack, depression) which may or may not be related to device use
Clinical care	Any contact with health services, for example: • A&E visit • Any hospital admission • Unexpected or unscheduled contact with clinical services (e.g. GP visits, contact with keyworker) Routine contact (e.g. as part of a care plan) would not normally count as an adverse event Any increase in existing treatment or any new treatment Any reduced adherence to treatment (does not include adherence to the app under-study), for example: • Reduces or stops taking prescribed medication • Reduces or stops scheduled visits to physician or therapist
Harmful behaviours	Any behaviour that results in physical or psychological harm to themselves or someone else, or puts themselves or others in danger, for example: • Self-harm or suicidal thoughts or behaviours • Aggressive behaviour towards others • Overly trusting or uncharacteristically risky behaviours • Negatively impacted personal relationships • Using device while driving • Using device to access harmful online sites
Intervention content /Format	Any negative reaction (other than symptom exacerbation/ deterioration coded above) to the device content or format of presentation, for example: • Dislike or irritation with visual, audio or gamification aspects of device
Assessment related	Any negative effects arising from study assessments (irrespective of device use), for example: • stress arising from the burden or content of assessments • anxiety about study arm allocation
Device deficiency	Any technical malfunction related ONLY to the device itself (e.g. “hangs”, doesn’t function correctly, login problems)
Technical malfunction	Technical malfunction NOT related to the device but IS related to study procedures (e.g. problems with device hardware such as handset or iPad, internet, study portals) Any general technical malfunction UNRELATED to the study device OR study procedures (e.g. televisions, laptops, and computers not used for the study)
Data breach/privacy	• Device accessed by unauthorised others (e.g. sharing or losing login details • Personal details from the device accessed by unauthorised others
Practical burden	• High or unexpected costs (e.g. phone/ internet bills) • Significant loss of time (e.g. due to device use)
Somatic/physical effects	• Eyestrain • Fatigue • Sleep disturbances • Headache
Adherence and response	Negative effects, not coded elsewhere, arising from lack of treatment adherence or response to the device, for example: • Missed or incomplete sessions • Perceived/actual non-response to treatment • Practical barriers to accessing the device (that cannot be attributed to device deficiency or technical malfunction) such as forgetting PIN, losing the device
Other	Any other negative experiences or events not covered above

Some of the categories such as technical malfunction might be less clear to clinicians, as they do not directly relate to the therapeutic component of the device. When assessing adverse events (AEs), it is essential to evaluate the entire device, not just the treatment component. This includes potential risks from using any mobile app. Furthermore, the MHRA approval mandates monitoring all aspects of the approved research for safety, covering study procedures, intervention and the device. One learning that came out of discussions during the development of the AE framework with other professionals was the need to have a separate AE category for “device deficiency” that is distinct from “technical malfunction”. Device deficiency is defined as “an inadequacy of a medical device related to its identity, quality, durability, reliability, safety or performance, such as malfunction, misuse or use error and inadequate labelling” [34]. This differentiation was highlighted by some of the academics on the team with experience in other DMHIs, to align with the requirements and terminology used by the regulatory framework.

#### Proposed safety plan for the trial

The trial is still ongoing at the time of writing and a full report of the STOP safety evaluation will be published as part of the trial outcomes. Here we present the tools we developed to aid STOP safety data collection, as described in the “Methods” sections of this paper. We also outline the final STOP safety analysis plan, which was subject to rigorous review and revision as part of the regulatory approval process.

##### Safety data collection

The Adverse Events Checklist used by researchers to proactively collect fortnightly (or weekly for more vulnerable participants) AE data is presented in Appendix G. Participants were asked each prompt question in turn to identify and record details of any adverse that had happened since the last researcher contact. For every event recorded researchers completed the remaining columns of the checklist to record a free text event description and to determine its seriousness, relatedness, expectedness and severity. The checklist will be administered every week rather than fortnightly with high-risk patients to mitigate any risks. It is likely that administering the checklist in these patients more than the rest might lead to a higher number of reported AEs. This will be taken into account in the analyses using sensitivity analyses.

##### Safety data analysis

The complete statistical analysis plan (SAP) for the STOP trial underwent a number of iterations in review with the regulators before approval was achieved. In terms of safety specifically, the approved plan included the following. Any AE/SAE involving the target clinical symptoms (paranoia) will be analysed separately from other AE/SAEs, due to the assessed (small) likelihood that the device could trigger these symptoms. This risk was singled out for separate analysis because it was the one of most concern to clinicians and regulators. Formal statistical analyses are unlikely due to small numbers of observations but the incidence rate of AEs (total number of those having the event divided by the person-months at risk) and the ratio of incidence rates of AEs between the two treatment arms per time period will be reported to allow detection of any safety concerns within the treatment arm.

Analysis of the checklist data will produce a list of adverse events along with frequencies, seriousness, relatedness and possible methods of prevention/mitigation. Additionally, demographic and clinical characteristics of those who experienced adverse events will identify patients who might be at a higher risk. Comparative statistical tests will be used to analyse the NEQ data between the treatment and control arms using a linear regression approach.

An overview of the pathway followed by the STOP team from start to finish is provided in Table 4. This shows the purpose of each step, some brief details on what it included and pointers allowing the reader to more easily navigate to relevant sections of the present paper and associated resources.

**Table 4:** One pathway for achieving an MHRA Notification of No Objection

Steps	Purpose	Sub-steps	Key relevant sections of this paper
**1. Is regulatory approval needed?**	Avoid unnecessary submission to MHRA	Check exemptions including: • single institution exemption • well-being as sole intended purpose • already UKCA/CE marked	• Introduction • Discussion
**2. Establish the current evidence on device safety and use/experience**	Quantify the “**benefit”** portion of the submitted case (“benefits outweigh the risks”)	Analyse and present any available data around the device safety, use/experience	• “Feasibility Study” from Method & Results in Section A^a^ • Supplementary File 1
		Conduct literature reviews of equivalent or similar devices, techniques or procedures	• “Literature searches” from Method & Results in Section A • Appendix B, C, E, F
**3. Develop a Risk Management Plan**	Quantify the “**risk”** portion of the submitted case (“benefits outweigh the risks”)	Identify all possible hazards and their corresponding harms	• “Hazard Identification” from Risk Management Plan under Method & Results in Section A • Appendix F
		Conduct a hazard analysis	• “Hazard Analysis” from Risk Management Plan under Method & Results in Section A • Tables 1 and 2
		Devise risk control strategies and calculate a new post-mitigation score	• “Risk Control and Re-evaluation” under Method in Section A • Appendix G
**4. Develop a “Clinical Investigation Plan” (as part of a wider “Clinical Evaluation Plan” for the product)**	Demonstrate how safety will be given priority in the proposed work Show how adverse events will be measured and assessed for relatedness to the research procedures, intervention or device	Develop a safety data **collection** plan	• “AE classification framework development” under Method in Section B^b^: • Table 3 • “Safety Data Collection” under “Proposed safety plan for the trial” Method & Results in Section B • Appendix D • Appendix G
		Develop a safety data **analysis** plan	• “Safety Data Analysis” under **“**Proposed safety plan for the trial”, Results in Section B • Appendix D

^a^ “Demonstrating the existing safety of the DMHI”

^b^ “Evaluating the safety of a DMHI within the proposed research study”

## Discussion

This paper details the steps that the STOP study team took to thoroughly assess the safety of a DMHI and achieve regulatory approval to conduct an RCT (MHRA’s NoNO). The example shared in this paper serves as a guide for academics and other professionals in the field. It provides a roadmap for the essential prerequisites, requirements and expectations regarding safety when seeking regulatory approval to conduct research with DMHIs. A fuller understanding of this pathway will significantly benefit research teams, clinicians and developers involved in the process of developing and delivering novel DMHIs.

**Fig. 1:**
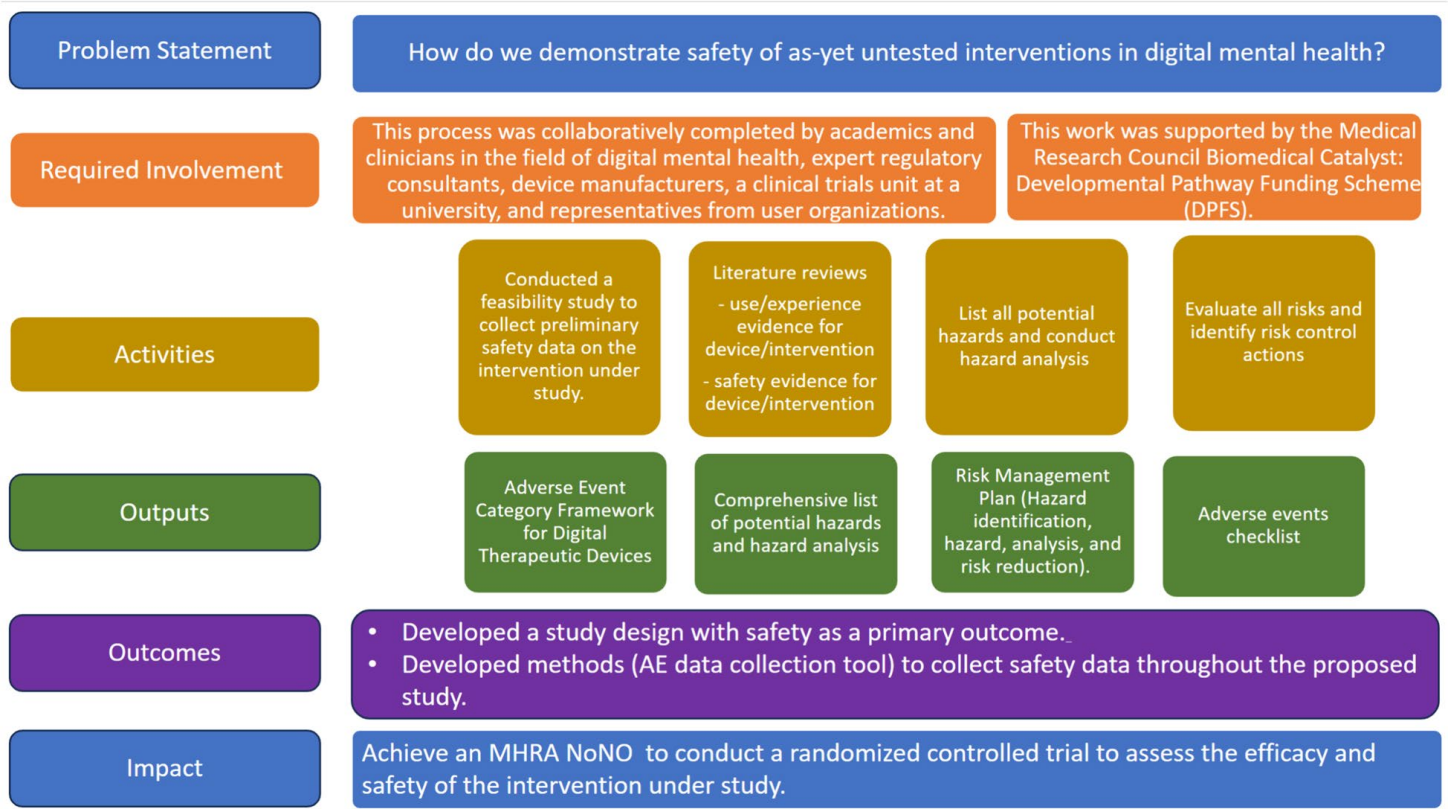
The process model of “How to demonstrate the safety of as-yet untested DMHI?

There are various key concepts and practical takeaways outlined in this paper. The overarching requirement is to compile an evidence-based argument that the benefit of the proposed device outweighs its risk to users, and this can only be done convincingly by the fullest consideration and quantification of that risk. The process by which one might do this can be broken down into various discrete steps. Figure 1 demonstrates the process model presented in this paper.

First, it is important to establish the safety of the DMHI even before testing its efficacy. This could be done by looking at the safety data of “equivalent” interventions that have been used in a similar population, studying the literature and/or conducting a feasibility/pilot study to assess the preliminary safety of the intervention. It is noteworthy that in the UK devices exclusively developed and used (either clinically or for research) within a single institution are exempt from formal regulatory approval requirements [35] which can provide an appropriate setting for gathering early-stage safety data. Second, conducting a comprehensive risk analysis specific to each DMHI is crucial [36]. This involves identifying all the potential hazards that are relevant to that DMHI, assessing any potential harm (likelihood and severity), calculating a risk rating per identified hazard, implementing risk control measures, reassessing risk, calculating a final post-risk score, denoting and reporting any residual risk and finally demonstrating that the expected benefits outweigh the identified risks in a quantifiable manner. Third, the safety of a new and untested DMHI needs to be evaluated as a primary outcome within the proposed research. It needs to hold the same importance as efficacy/effectiveness, irrespective of the academic research agenda. A safety evaluation plan needs to be integrated within the study protocol or presented separately as a standalone study.

Fourth, a helpful component of any safety evaluation is the use of a framework for organising the data to be collected, given the likely breadth of possible adverse events. The Adverse Event Category Framework for Digital Therapeutic Devices provides one such possibility. At a more practical level, this must be supplemented by a structured approach to collecting and evaluating individual adverse events. The Adverse Events Checklist (provided in appendices) received regulatory approval for use in the STOP trial and could usefully serve as a guide for others. By incorporating categorisation of each entry on the key dimensions of seriousness, severity, relatedness and expectedness, it allows a research team to more easily demonstrate their intended compliance with reporting requirements. It also facilitates gathering a richer dataset around negative effects that will go on to permit a more comprehensive analysis than previous traditional practices [11]. A scoping review on the recording of harms in RCTs of behaviour change interventions has mapped out the categories of harms found in that literature [35]. As might be expected, there is some overlap with our AE category framework, such as physical and psychological harms, which is reassuring and validatory. In contrast, group-level harms (such as the impact of a behaviour change intervention on culture, environment or health equity) feature strongly in the scoping review but are absent from our framework, which focused exclusively on individual participant-level harms data. It will be important for future studies to consider whether macro-level harms relevant to behaviour change interventions might also be relevant to DMHI interventions.

It is important to highlight the time involved in the processes summarised in this paper. In the present worked example acquiring regulatory approval (MHRA NoNO) took approximately 6 months and the authors’ recommendation is to allow a timeframe of up to 9 months, if working from a position of relatively little prior knowledge and experience. This timeline is necessary to allow for the involvement of clinical and technical experts, patient groups and regulatory consultants. In the present worked example employing a regulatory consultant played a vital role in ensuring compliance with all regulatory requirements and smooth passage through regulatory review. Their knowledge of the complex regulatory landscape provided a key interface between software developers and the academic team to ensure that the requisite information was compiled and presented in a manner compliant with the appropriate national and international standards [37]. Academic teams are advised to routinely cost such expertise into research projects involving medical devices, unless equivalent institutional support is already available.

## Limitations

It is important to be aware that this paper provides an example of how *one* DMHI assessed safety and achieved regulatory approval. The experiences of other DMHIs will most likely differ. Thus, it is important to view this process flexibly and adapt it to each DMHI. Furthermore, this example is UK-centric. Even though the process described might be helpful for DMHIs applying for regulatory approval outside the UK, professionals need to be aware of the needs of their specific regulatory environment.

## Conclusion

The example provided in this paper can be adapted by other professionals in the digital mental health field to help them navigate complex regulatory processes. Prioritising and emphasising safety and regulatory compliance allows researchers to contribute to the responsible development of DMHIs. Ensuring that the benefit of these interventions outweighs any risks that they carry is important for building confidence and trust among clinicians, patients and academics. The systematic approach to safety evaluation outlined here sets a valuable precedent for assessing the safety of DMHIs.

## Supplementary Information

Supplementary Material 1.

Supplementary Material 2.

## Data Availability

All data generated or analysed during this study are included in this published article in the form of tables and appendices.

## Abbreviations

AE: Adverse event
DMHI: Digital mental health intervention
ICH-GCP: International Council for Harmonisation of Technical Requirements for Pharmaceuticals for Human Use—Good Clinical Practice
MHRA: Medicines and Healthcare products Regulatory Agency
NEQ: Negative Effects Questionnaire
NHS: National Health Service
NICE: National Institute of Health and Care Excellence
NoNO: Notification of No Objection
PRISMA: Preferred Reporting Items for Systematic Reviews and Meta-Analyses RCT: randomised controlled trial
RCT: Randomised controlled trial
SAE: Serious adverse event
SaMD: Software as a Medical Device

## Footnotes

1 “*You can no longer use STOP as the product has been withdrawn. You will shortly be contacted by a member of the research team who will explain and offer further support if required. In the meantime, if you require more urgent assistance, please contact the study helpline at 020 784 80,425 or clinical support email at stopclinicalsupport@kcl.ac.uk”.*
