# Supplementary material for: Developing a process for assessing the safety of a digital mental health intervention and gaining regulatory approval: a case study and academic’s guide
Source: Trials. 2024 Sep 10;25:604. doi: 10.1186/s13063-024-08421-1 (PMC11385814; doi:10.1186/s13063-024-08421-1)
Supplement: Supplementary file 1 — Supplementary Material 1. [file 13063_2024_8421_MOESM1_ESM.docx]

**Research question: Does taking CBM-pa sessions have any significant effects on short term mood?**

**Main outcome paper**: Yiend J, Lam CLM, Schmidt N, et al. Cognitive bias modification for paranoia (CBM-pa): a randomised controlled feasibility study in patients with distressing paranoid beliefs. Psychological Medicine. 2023;53(10):4614-4626. [doi:10.1017/S0033291722001520](https://www.cambridge.org/core/journals/psychological-medicine/article/cognitive-bias-modification-for-paranoia-cbmpa-a-randomised-controlled-feasibility-study-in-patients-with-distressing-paranoid-beliefs/2563E17112B7984FC28CE26E4ACB2437#article)

**Brief summary of the trial and aims**

The ‘Cognitive Bias Modification for paranoia’ (CBM-pa) study is a feasibility, double-blind, randomised controlled trial (RCT) for stabilised outpatients with persistent, distressing paranoid symptoms. The target was to recruit 60 patients and randomize them into intervention (CBM-pa) and control arm. Both researchers and participant should be masked from group assignment and should therefore be blinded for the duration of the study. Patients will be randomised at a 50:50 ratio, to computerised CBM-pa or a text-reading control intervention, receiving one 40-min session per week, for 6 weeks. CBM-pa involves participants reading stories on a computer screen, completing missing words and answering questions about each story in a way that encourages more helpful beliefs about themselves and others. The cognitive mechanism targeted by CBM-pa is biased interpretation of emotional ambiguity (forthwith, ‘interpretation bias’). Treatment as Usual will continue for patients in both groups. Patients will be assessed by a researcher blind to allocation, at baseline, each interim session, post treatment and 1- and 3-month follow-up post

**Method**

Spontaneously reported adverse events and serious adverse events were recorded throughout the trial.

In addition, Visual Analogue Scales were used in which participants answer ‘How do you feel right now?’ by placing a mark on a 100 mm line. Four separate scales are marked corresponding to ‘Anxious’, ‘Sad’, ‘Paranoid’ and ‘Friendly’ respectively. Each line has anchor points ‘not at all’ (0) and ‘completely’ (100), which yield scores that represent a % value. VAS scales were given before and after sessions 2, 3, 4 and 5, which correspond to the study’s interim timepoints, and change scores were calculated by subtracting pre from post session values. Positive values therefore reflect an increase in the measured state mood. Scales were not used at baseline or any of the main outcome timepoints to avoid overburn of participants and contamination of main study outcomes. These time points are not needed to address the research question.

**Results**

Table S1 shows mean differences between allocated treatment arm, and statistical test results.

**Table S1**: Mean difference between trial arms (2= Intervention, 1= Control) at each interim timepoint (2, 3, 4, 5). Values use to calculate the mean difference are themselves change scores on the relevant VAS scale (anxiety, sadness, paranoia and friendliness) from pre- to post- session. A negative mean difference indicates that the intervention group has shown a bigger pre- to post- session drop in mood than the control group . For example, reduction in anxious mood across session 2 is around 10% more in the intervention group than the control group (row 1).

| Variable | Arms | Time | Mean difference | (95% C.I) | z | p |
| --- | --- | --- | --- | --- | --- | --- |
| VAS scales (post minus pre) |  |  |  |  |  |  |
| Anxious mood change | 2 vs 1 | 2 | -10.961 | (-20.244 to -1.677) | -2.314 | 0.021 |
| Anxious mood change | 2 vs 1 | 3 | 3.179 | (-6.103 to 12.461) | 0.671 | 0.502 |
| Anxious mood change | 2 vs 1 | 4 | -2.715 | (-12.074 to 6.643) | -0.569 | 0.57 |
| Anxious mood change | 2 vs 1 | 5 | -9.904 | (-19.272 to -0.537) | -2.072 | 0.038 |
| Sad mood change | 2 vs 1 | 2 | -6.156 | (-15.514 to 3.201) | -1.29 | 0.197 |
| Sad mood change | 2 vs 1 | 3 | -0.807 | (-10.164 to 8.55) | -0.169 | 0.866 |
| Sad mood change | 2 vs 1 | 4 | -1.322 | (-10.759 to 8.115) | -0.275 | 0.784 |
| Sad mood change | 2 vs 1 | 5 | 0.459 | (-8.989 to 9.906) | 0.095 | 0.924 |
| Paranoid mood change | 2 vs 1 | 2 | -10.996 | (-18.711 to -3.281) | -2.793 | 0.005 |
| Paranoid mood change | 2 vs 1 | 3 | -5.057 | (-12.772 to 2.658) | -1.285 | 0.199 |
| Paranoid mood change | 2 vs 1 | 4 | -3.290 | (-11.07 to 4.491) | -0.829 | 0.407 |
| Paranoid mood change | 2 vs 1 | 5 | 3.980 | (-3.809 to 11.77) | 1.002 | 0.317 |
| Friendly mood change | 2 vs 1 | 2 | -1.200 | (-10.604 to 8.204) | -0.25 | 0.803 |
| Friendly mood change | 2 vs 1 | 3 | 1.833 | (-7.571 to 11.237) | 0.382 | 0.702 |
| Friendly mood change | 2 vs 1 | 4 | -6.517 | (-16.002 to 2.968) | -1.347 | 0.178 |
| Friendly mood change | 2 vs 1 | 5 | 5.605 | (-3.891 to 15.101) | 1.157 | 0.247 |

Figure S1:

Dose x Arm interaction: p=0.07 Dose x Arm interaction: p=0.76:

Significant difference at Dose 4 (p=0.04) No significant pairwise differences

Dose x Arm interaction: p=0.04: Dose x Time interaction: p=0.34

Significant difference at Dose 1 (p=0.005) No significant pairwise differences

**Conclusions**

The intervention was deemed safe in so far as there were no observed adverse effects on short term mood in the intervention compared to the control group while undertaking the sessions. There was some evidence of short term mood benefits in the intervention group compared to control.
