# Supplementary material for: Developing a process for assessing the safety of a digital mental health intervention and gaining regulatory approval: a case study and academic’s guide
Source: Trials. 2024 Sep 10;25:604. doi: 10.1186/s13063-024-08421-1 (PMC11385814; doi:10.1186/s13063-024-08421-1)
Supplement: Supplementary file 2 — Supplementary Material 2. [file 13063_2024_8421_MOESM2_ESM.docx]

**Appendix A**

**The full list of participants/contributors in alphabetical order**

| **Name** | **Affiliation** | **Risk Management Plan** | **AE Classification Framework: expert consultation panel** |
| --- | --- | --- | --- |
| Andrew Gumley | STOP trial Data Monitoring Committee chair; Statistician |  | x |
| Neha Gupta | Avegen, Manufacturer | x |  |
| Che-Wei Hsu | STOP trial Clinical Psychologist | x |  |
| Pamela Jacobsen | STOP trial Co Investigator, Clinical Psychologist | x | x |
| Thomas Kabir | The McPin Foundation | x |  |
| Nayan Kalnad | CEO Avegen, Manufacturer | x |  |
| Alex Kenny | The McPin Foundation | x |  |
| Jeroen Keppens | STOP trial Co Investigator, Academic |  | x |
| Philip McGuire | STOP trial Co Investigator, Psychiatrist | x | x |
| Caroline Murphy | King's Clinical Trials Unit |  | x |
| Emanuelle Peters | STOP trial Co Investigator, Clinical Psychologist | x | x |
| Sumiti Saharan | Avegen, Manufacturer | x |  |
| Sukhi Shergill | STOP trial Co Investigator, Psychiatrist | x | x |
| Carolina Sportelli | Avegen, Manufacturer | x |  |
| Daniel Stahl | STOP trial Co Investigator, Statistician | x | x |
| Chris Taylor | Data monitoring committee member, Clinical Psychologist |  | x |
| Ben Wensley Stock | Regulatory consultant | x | x |
| Jenny Yiend | STOP trial Chief Investigator, Academic | x | x |

**Appendix B**

**Literature review methodology**

**Literature review 1:**

The aim of the first literature review was to answer the research question “What evidence exists of **use or experience** of this device or other closely related digital therapies such as ones that use cognitive bias modification, in clinical paranoia and psychosis samples?” (assessing usability and acceptability as requested by the MHRA^39^). Five scientific databases were used: CINAHL, PubMed, EMBASE, PsycINFO, and Medline. The search was conducted on 03/23/2022 by R.T. There was no limit on dates. Articles that were written in English and addressed the effectiveness of a digital mental health intervention in reducing paranoid/psychosis symptoms in a clinical sample were included. These were the search terms used:

**Search terms for literature review 1:**

1. (Cognitive Bias Modification for interpretation).ti,ab

2. (CBM-I).ti,ab

3. (digital intervention).ti,ab

4. (psychosis).ti,ab

5. (paranoia).ti,ab

6. (1 OR 2 OR 3)

7. (4 OR 5)

8. (6 AND 7)

The included studies were appraised using the clinical data appraisal tools (See Appendix C).

**Literature review 2:**

The aim of the second literature review was to answer the research question “What evidence exists of **the safety of this or similar devices** in clinical paranoia and psychosis samples?”. Five scientific databases were used: CINAHL, PubMed, EMBASE, PsycINFO and Medline. The search was conducted on 06/04/2022 by R.T. There was no limit on dates. Articles that were written in English and addressed the safety of a digital mental health intervention in reducing paranoid/psychosis symptoms in a clinical sample were included. These were the search terms used:

**Search terms for literature review 2:**

1 digital.ab,ti.

2 online.ab,ti.

3 mental health.ab,ti.

4 therapy.ab,ti.

5 paranoia.ab,ti.

6 psychosis.ab,ti.

7 risk.ab,ti.

8 "negative effect*".ab,ti.

9 harm.ab,ti.

10 adverse event.ab,ti.

11 "safe*".ab,ti.

12 1 or 2

13 3 or 4

14 5 or 6

15 7 or 8 or 9 or 10 or 11 16 12 and 13 and 14 and 15

The included studies were appraised using the clinical data appraisal tools (See Appendix C).

**Appraisal of clinical data on effectiveness tool**

| **Suitability criteria** | **Description** | **Grading system** |
| --- | --- | --- |
| Appropriate device | Were the data generated from the device in question? | D1 -Actual device  D2 – comparable device (digital cognitive intervention that is aimed at people struggling with paranoia or psychosis)  D3 – other medical device |
| Appropriate device application | Was the device used for the same intended use (e.g. methods of deployment, application)? | A1 – same use  A2 – minor deviation  A3 – major deviation |
| Appropriate patient group | Were the data generated from a patient group that is representative of the intended treatment population (e.g. age, sex) and clinical condition (i.e. disease, including state and severity)? | P1 – Applicable  P2 – Limited  P3 – Different population |
| Acceptable report/data collation | Do the reports or collations of data contain sufficient information to be able to undertake a rational and objective assessment? | R1 – High quality  R2 – minor deficiencies  R3 – insufficient  information |

Note: *To assess suitability the more level 1 grades, the greater the weight of evidence.*

**Appraisal of clinical data on safety**

| **Data contribution criteria** | **Description** | **Grading system** |
| --- | --- | --- |
| Data source type | Was the design of the study appropriate? | T1 – yes  T2 – no |
| Outcome measures | Do the outcomes measures reported reflect the intended performance of the medical device? | O1 – Yes  O2 – No |
| Follow up | Is the duration of follow-up long enough to assess whether duration of treatment effects and identify complications? | F1 – yes  F2 – no |
| Statistical significance | Has a statistical analysis of the data been provided and is it appropriate? | S1 – yes  S2 – no |
| Clinical significance | Was the magnitude of the treatment effect observed clinically significant? | C1 – yes  C2 – no |

Note: *To assess suitability the more level 1 grades, the greater the weight of evidence.*

**Appendix C**

**How AE seriousness, severity, relatedness and expectedness were defined**

| **Seriousness** | According to the Health Research Authority (HRA)^35^ a serious AE (SAE) is very specifically defined to include anything that was, or could have been, life threatening ^40^ . This serves as a starting point from which study teams are expected to articulate their own unique SAE definition that should be included in the study protocol. In the case of the STOP trial, SAEs were defined as an AE that:   - results in death - is life-threatening* (i.e. at-risk of death) - requires hospitalisation or prolongation of existing hospitalisation - results in persistent or significant disability or incapacity - jeopardises the patient, or requires intervention to prevent one of the outcomes listed above   *Life-threatening, by definition, refers to an event in which the subject was at risk of death at the time of the event; it does not refer to an event which hypothetically might have caused death if it were more severe. |
| --- | --- |
| **Severity** | Severity is decided based on the impact that the event has on the patient. In STOP, the team categorised severity at one of three levels: mild, moderate and severe. Decisions on the severity category given to each AE are made collaboratively between the researcher and the participant based on the level of impact the AE has on the participant. |
| **Relatedness** | Relatedness refers to the likelihood or possibility that the AE is caused by the intervention under study or simply as a result of taking part in the trial itself. In STOP, relatedness was assessed by researchers on a case-by-case basis by gathering more information (usually direct from the participant) about the circumstances surrounding the event and ascertaining the participants’ own opinion on a causal link. In straightforward instances, the final decision on relatedness is made by the researcher, however, if unclear this would be escalated to the chief investigator for support. |
| **Anticipated or not anticipated (Expectedness)** | In a pharmacological trial whether an AE is anticipated or not is decided by referring to the drug’s manufacturer documentation to see if such events were anticipated by the manufacturer^8^. However, for non-pharmacological trials this does not apply. Thus, for the STOP trial the study team had to develop a comprehensive list of anticipated adverse events relevant to the current trial. The finalised list of anticipated AEs was cross checked against the list of harms in the Risk Management Plan to ensure that all harms could be accurately placed within one of the existing categories. That being the case, no changes were needed to the preliminary framework for the STOP trial (although in other trials it is conceivable that the particular harms identified could lead to the need for new categories specifically related to the intervention or its target health concern). The resultant list of categories and example events served as the definition of the trial’s “anticipated” events. |

**Appendix D**

**PRISMA Flowcharts**

**Literature review 1:**


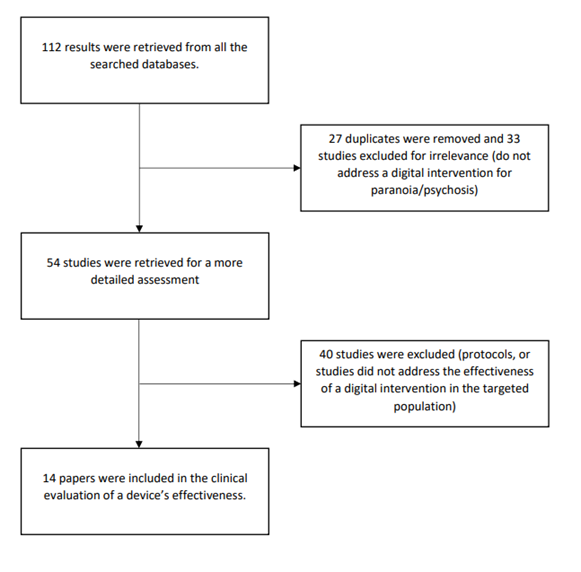


**Literature review 2:**
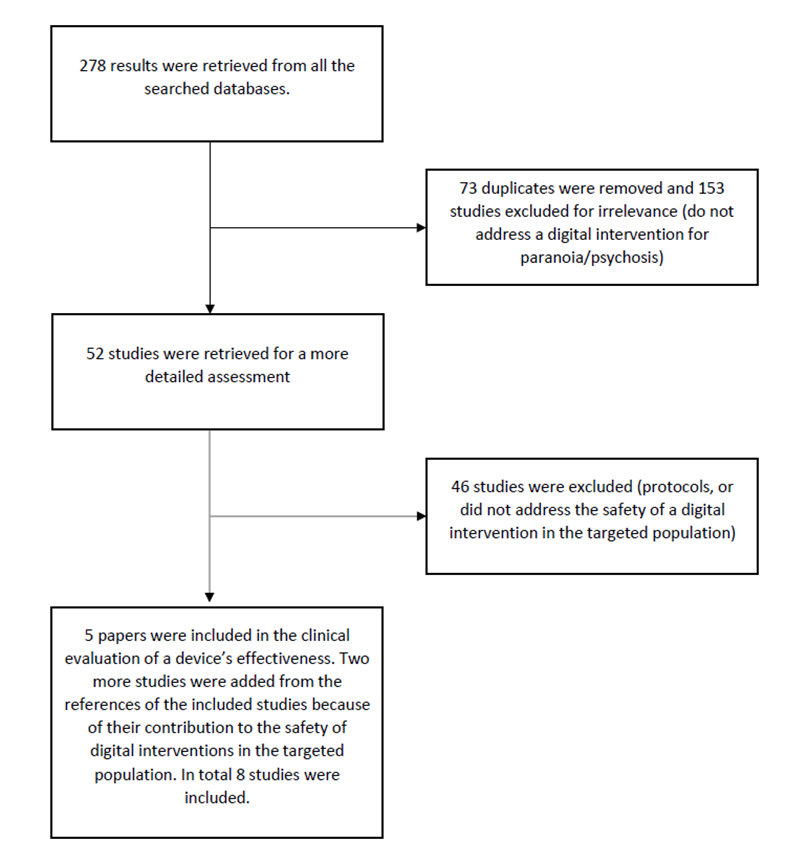


**Appendix E**

**The list of identified hazards and corresponding harms**

|  | **Identified Hazard for STOP** | **Corresponding Harm** |
| --- | --- | --- |
| **1** | Screen Flicker | Potential to Increase Paranoia for a transient period during use. |
| **2** | Software fails preventing use.   Device not used. | Reduced treatment effectiveness. |
| **3** | User entries are marked incorrectly by the software | Exacerbate existing paranoid symptoms |
| **4** | Software Crash   Device not used. | Reduced treatment effectiveness. |
| **5** | Being exposed to potentially triggering therapy content   (Engaging in psychological therapy) | Exacerbate existing paranoid symptoms |
| **6** | User receives negative feedback in the device (e.g. believe that they have given the wrong answer) | Short-term deterioration of mood. |
| **7** | Use of a connected digital medical device in paranoid population | Patient believes that the app is being used against them and exacerbates their disorder.   Exacerbate existing paranoid symptoms |
| **8** | Scenario described in app is encountered in real life outside of the product use | Short-term increase in risk of increased paranoia or negative emotions   Exacerbate existing paranoid symptoms |
| **9** | Emotional or triggering content (e.g. reading questions about symptoms, therapeutic content) | Short-term deterioration of mood. |
| **10** | Engaging in psychological therapy | Exacerbate existing paranoid symptoms |
| **11** | User experiences too much screen time when using the app alongside other normal screen usage | Short-term eyestrain, fatigue or headache |
| **12** | Engaging in self-administered therapy   Engaging in Psychological treatments without direct clinician involvement | Patient experiences new symptoms |
| **13** | Losing the handset   Device not used. | Reduced treatment effectiveness. |
| **14** | Losing access to the app   Device not used. | Reduced treatment effectiveness. |
| **15** | Nonadherence to STOP treatment | Reduced treatment effectiveness. |
| **16** | User confusion about device sessions treatment procedures.   Lack of adherence to device treatment, | Reduced treatment effectiveness. |
| **17** | Therapeutic effect acts by encouraging more trusting attitudes and interpretations and is not picked up and managed by required existing clinical engagement and treatments | Subject becomes overly trusting/ less risk averse leading indirectly to potential harm. |
| **18** | Cognitive load too high for subject | Short-term fatigue or boredom.   Reduced treatment effectiveness. |
| **19** | Patient's use of the app becomes known by others (e.g. intentional or unintentional sharing of PIN)   Data/Privacy Breach. | Distress/ embarrassment and potential deterioration of mood.   Deterioration of mood |
| **20** | User’s personal/health condition data becomes known to someone with access to their phone via push notifications generated by STOP   Data/Privacy Breach | Distress/ embarrassment and potential symptom exacerbation.   Exacerbation of Symptoms. |
| **21** | Users phone is hacked   Data/Privacy Breach | Distress/ embarrassment and potential symptom exacerbation.   Exacerbation of Symptoms. |
| **22** | The audio that accompanies some scenarios of the intervention | User startled but no harm.   Deterioration of mood. |
| **23** | Content style not enjoyed by the subject | User Irritation and potentially reduced effectiveness of device therapeutic effect very minimal potential for exacerbation of symptoms.   Deterioration of mood. |
| **24** | User finds the content in the sessions patronising and/or irritating | User irritation and potentially reduced effectiveness of device therapeutic effect very minimal potential for exacerbation of symptoms. |
| **25** | User stops or reduces existing pharmacological treatment due to taking STOP, without seeking clinical advice first   Reduced adherence to existing clinical treatments. | Mental health condition deteriorates |
| **26** | User stops or reduces existing face to face clinical care/ treatment   Reduced adherence to existing clinical treatment. | Mental health condition deteriorates |

**Appendix F**

**Final STOP Hazard Analysis**

| **Identified Hazard** | | **Preliminary Hazard Analysis** | | | **Risk Control** | **Re-Evaluation (Post Risk Control)** | | |
| --- | --- | --- | --- | --- | --- | --- | --- | --- |
|  |  | **Probability** | **Severity** | **Risk Score** |  | **Probability** | **Severity** | **Risk Index** |
| **1** | Screen Flicker | 2 | 1 | 2 | Optimise the STOP device to prevent screen flicker as a design control requirement.   Validation reports must confirm that the STOP device doesn't exhibit screen flicker.   Researchers conduct regular semi-structured interview phone calls with users in the study to detect adverse events, including screen flicker instances.   The product must include a user issue reporting and technical support feature accessible via the provided tech support email.   An app feature should include a clinical contact email address in the device’s electronic help section.   Mood monitoring occurs before and after each session; if screen flicker negatively impacts mood or other measures, an alert is sent to the researcher for follow-up. Hazard analysis modelling correctness is verified, and any issues are documented as problem reports.   Users experiencing paranoia due to screen flicker can report through the study helpline, clinical support email, or during regular interview calls. | 1 | 1 | 1 |
| **2** | Software fails preventing use.   Device not used. | 6 | 1 | 6 | Instructions for Use (IFU) must ensure:   Software is up to date.  Users check device battery and charge status.  Helpline details in printed IFU and app's help feature.   Regular bi-weekly calls for study insights and enhancements.   Develop Post Market Surveillance for app crash reports, aiding improvement.   Final product includes Post Market Surveillance to identify enhancements. | 2 | 1 | 2 |
| **3** | User entries are marked incorrectly by the software | 6 | 2 | 12 | A requirement to validate the responses to questions was validated by QA. | 2 | 2 | 4 |
| **4** | Software Crash   Device not used. | 6 | 1 | 6 | Software validation for identifying coding errors and product verification and validation according to ISO 62304 standards.   Instructions for Use (IFU) requirements include ensuring the latest software version, device battery check, and charging instructions.   Helpline contact provided in printed IFU for user support.   During clinical investigation, regular calls every 2 weeks with researchers to assess solution stability and gather insights for enhancements.   Post-Market Surveillance aimed at identifying faults for potential product improvements in the final version. | 3 | 1 | 3 |
| **5** | Software fails preventing use   Device not used. | 5 | 1 | 5 | 1.Validate software for coding issues and verify products according to ISO 62304.  2.Instructions must guide users to update software, check battery, and charge the device.  3.Include helpline details in the hard copy user instructions.  4.Researchers oversee session booking, addressing scheduling issues with manufacturer during Clinical Investigations.  5.Scheduled calls every 2 weeks by researchers during the study for insights and solution stability.  6.The final product will have Post Market Surveillance tools for fault detection and product enhancement. | 3 | 1 | 3 |
| **6** | Being exposed to potentially triggering therapy content (Engaging in psychological therapy) | 6 | 2 | 12 | 1.Device prescriber function required for ongoing clinical treatment.  2.Product feature allows prescriber/investigator to recall use if treatment stops.  3.Researchers conduct fortnightly (or weekly for some) semi-structured interviews with users, tracking adverse events and increased paranoia.  4.Mood monitored before and after STOP device sessions in clinical investigations.  5.Automated alerts sent to researchers if mood worsens across three sessions; follow-up call to check well-being.  6.Users can report worsened paranoia through study helpline, clinical support email, or during interviews. | 3 | 2 | 6 |
| **7** | User receives negative feedback in the device (e.g. believe that they have given the wrong answer) | 4 | 1 | 4 | The requirements for ongoing clinical treatment and oversight include a feature enabling clinical investigators to recall the product from subjects if treatment discontinues. The product should feature mood or paranoia deterioration detection over three sessions, triggering automated alerts to clinical prescribers for follow-up. Researchers must monitor mood before and after sessions in STOP devices during clinical investigations. The product must facilitate direct user reporting through the 24-h helpline, clinical support email, and scheduled interview calls. Throughout the investigation, researchers conduct semi-structured interview calls with users every fortnight (or weekly for those more susceptible) to inquire about adverse events since the last contact. | 2 | 1 | 2 |
| **8** | Use of a connected digital medical device in paranoid population | 4 | 3 | 12 | The requirements for ongoing clinical treatment and oversight include a feature allowing clinical oversight/clinical investigation researchers to recall the product if treatment discontinues. A product feature should detect mood or paranoia worsening over three consecutive sessions, triggering an automated alert to the clinical prescriber/researcher for follow-up. Mood monitoring before and after each STOP device session is mandatory within clinical investigations. The product should allow users to report concerns via the 24-h study helpline, clinical support email, or during scheduled interview calls with the research team. Semi-structured interview calls are conducted fortnightly (weekly for more susceptible individuals) throughout the investigation, addressing any adverse events since the last contact. | 3 | 3 | 9 |
| **9** | Scenario described in app is encountered in real life outside of the product use | 2 | 3 | 6 | 1.A requirement for ongoing clinical treatment and oversight, with the ability to recall the product from subjects if treatment discontinues.  2.A feature to detect deteriorating mood, paranoia, or other scales across three sessions, triggering an automated alert to the clinical prescriber. The prescriber contacts the participant for follow-up.  3.Mood monitoring before and after each session within STOP device clinical investigations.  4.User instructions enabling direct concern reporting through a 24-h helpline, clinical support email, or scheduled interviews during investigations.  5.Researchers conduct semi-structured interview calls with users every fortnight (or weekly for those more susceptible) throughout the clinical investigation, addressing adverse events since the last contact. | 2 | 2 | 4 |
| **10** | Emotional or triggering content (e.g., reading questions about symptoms, therapeutic content) | 3 | 3 | 9 | 1.The product should have ongoing clinical oversight and the ability to recall it if needed.  2.The product must detect worsening mood or other scales across sessions and alert the clinical prescriber.  3.Mood is to be monitored by a researcher before and after each session.  4.Users can report concerns through the study helpline, clinical support email, or regular interviews.  5.Researchers conduct regular interviews with users to discuss any adverse events. | 1 | 2 | 2 |
| **11** | Engaging in psychological therapy | 5 | 2 | 10 | 1.The need for ongoing clinical treatment with oversight and the ability to recall the product if treatment discontinues.  2.A feature to detect worsening mood or paranoia over three sessions, triggering an alert to the clinical prescriber for follow-up.  3.Monitoring mood before and after sessions in the STOP device during clinical investigations.  4.Inclusion of user-friendly reporting methods for concerns, including helpline, email, and interview calls during investigations.  5.Researchers conducting regular interview calls with users to gather feedback, especially on adverse events, during clinical investigations. | 1 | 2 | 2 |
| **12** | User experiences too much screen time when using the app alongside other normal screen usage | 2 | 1 | 2 | 1.The STOP product feature requires users to have comfort breaks while aiming to complete a session in one go. Users need to press “continue” at each quarter point, but they can leave and return to sessions until the weekly completion window ends.  2.Risk control validation reports show that the STOP device allows comfort breaks and session interruptions without data loss.  3.PMS features are needed to monitor product usage and alert users and prescribers about excessive use.  4.During clinical studies, researchers conduct semi-structured interview calls every two weeks (or weekly for more susceptible individuals) to inquire about adverse events.  5.The product requires a feature and user instructions for direct reporting of concerns through the study helpline, clinical support email, and interview calls during investigations. a) the 24-h study helpline provided in the help section of the device  b) clinical support email provided in the help section of the device.   c) during clinical investigations, the fortnightly (weekly for those with elevated susceptibility) interview phone call with the research team | 1 | 1 | 1 |
| **13** | Engaging in self-administered therapy   Engaging in Psychological treatments without direct clinician involvement | 2 | 3 | 6 | 1. Need for ongoing clinical oversight, including the ability to recall the product if treatment discontinues.  2. Requirement for an automated alert system to notify clinicians about worsening mood or other factors after three consecutive sessions, leading to participant follow-up.  3. Continuous mood monitoring before and after sessions in the STOP device during clinical investigations.  4. Product features and user instructions allowing direct reporting of concerns through the 24-h study helpline, clinical support email, and regular interview calls with the research team.  5.Regular semi-structured interview phone calls with users during clinical investigations to assess adverse events and well-being. | 1 | 2 | 2 |
| **14** | Losing the handset   Device not used. | 6 | 2 | 12 | Users should be able to report a missing phone through the following channels:  a) Utilising the 24-h study helpline available in the device’s help section.  b) Contacting the clinical support email provided in the device’s help section.  c) During clinical investigations, participating users will have regular interview phone calls with the research team, conducted fortnightly (or weekly for those with elevated susceptibility).   Another essential product feature is the ability for prescribing clinicians or clinical investigation researchers to monitor users’ adherence to prescribed and scheduled usage. This feature should include notifications to alert the clinician or researcher in case of non-compliance. | 1 | 1 | 1 |
| **15** | Losing access to the app   Device not used. | 6 | 2 | 12 | 1.The need for a secure and dependable feature to reset user access credentials is essential.  2.During clinical investigations, researchers will hold semi-structured interview phone calls with users every 2 weeks (or weekly for more “susceptible” individuals). These calls will cover adverse events and identify login issues.  3.The technical support contact information should be included in printed labelling or other printed forms, including if the product shifts to an Instructions for Use (IFU) format. | 1 | 1 | 1 |
| **16** | Nonadherence to STOP treatment | 5 | 2 | 10 | 1.A feature is needed to send session reminders to users.  2.Another feature should identify non-compliance and send reminders.  3.A feature is required to identify non-adherence and notify the research team.  4.Adherence rates are continuously monitored through the analytics dashboard and reported to the Data Monitoring Committee for clinical investigations. | 3 | 1 | 3 |
| **17** | User confusion about device sessions treatment procedures.   Lack of adherence to device treatment, | 4 | 2 | 8 | 1.A necessary feature involves instructions and practice items within the product to guide users through sessions, assessments, and treatment steps.  2.The product also includes a feature that detects non-adherence, such as missed sessions or incomplete tasks, and sends notifications to the clinical research team.  3.In clinical studies, adherence rates are consistently tracked via the researcher app’s analytics dashboard and reported to the Data Monitoring Committee as a significant study milestone. | 1 | 1 | 1 |
| **18** | Therapeutic effect acts by encouraging more trusting attitudes and interpretations and is not picked up and managed by required existing clinical engagement and treatments | 1 | 4 | 4 | 1.The requirement for gradual exposure of users to scenarios, starting with phrases like “I imagine/think/sense” and progressing to “I believe/am sure/know”, may not reduce impact or likelihood. Suggest deletion as a design control. Scenarios should be hypothetical, not factual, to minimise paranoid responses when no danger signal is present.  2.Users should be instructed to endorse positive non-paranoid perspectives only in the context of the specific scenario, not in general.  3.Scenarios must undergo extensive review by clinicians and service users. Unsuitable items should be rephrased or replaced.  4.Additional support (weekly calls instead of fortnightly) should be provided to those identified as more “suggestible” to prevent overapplication of the therapeutic effect. | 1 | 3 | 3 |
| **19** | Cognitive load too high for subject | 3 | 1 | 3 | 1.The STOP product should allow users to take comfort breaks and resume sessions where they left off. Users need to press “continue” every quarter point, and they can leave and rejoin sessions until the weekly completion deadline (Friday midnight).  2.Validation reports confirm that STOP supports comfort breaks and session interruption without data loss.  3.PMS features must monitor usage and notify users and prescribers of excessive use.  4.Researchers conduct semi-structured interviews every two weeks (or weekly for susceptible users) to inquire about adverse events during clinical investigations.  5.Users can report concerns through the helpline, clinical support email, or scheduled research team interviews.  6.The app, IFU, and information leaflet must emphasise that the app complements, not replaces, usual treatment, and users should consult clinicians before altering treatment.  7.Users must be informed in materials about sharing symptom changes with the study team and clinicians.  8.New/worsening symptoms should be noted during adverse events checklists in appointments, and participants are encouraged to inform clinicians about changes. | 1 | 1 | 1 |
| **20** | Patient's use of the app becomes known by others (e.g., intentional or unintentional sharing of PIN). Data/Privacy Breach. | 5 | 2 | 10 | 1.The STOP device’s IFU and set pin screen should include warnings against sharing the security code.  2.Labels on the device should prompt users to contact support if they suspect data breaches.  3.Product feature and IFU instructions should enable users to report concerns via the 24-h study helpline, clinical support email, and research team interviews during clinical investigations. | 3 | 2 | 6 |
| **21** | User's personal/health condition data becomes known to someone with access to their phone via push notifications generated by STOP   Data/Privacy Breach | 6 | 3 | 18 | 1.Notification messages must be generic, devoid of personal data and health condition references, including paranoia.  2.Labels should prompt users to contact support if they suspect a breach of their private data.  3.Product feature and IFU’s user instructions must facilitate reporting concerns via: a) 24-h study helpline in the device’s help section, b) clinical support email in the device’s help section, c) during clinical investigations, the regular interview phone call with the research team, adjusted frequency for elevated susceptibility cases. | 1 | 1 | 1 |
| **22** | Users phone is hacked   Data/Privacy Breach | 6 | 3 | 18 | 1.Users must authenticate with a 4-digit PIN each time they access the app, as a design control.  2.Data stored in the device sandbox must be encrypted and can only be decrypted with a unique deciphering key, as a design control.  3.A product feature and user instructions (IFUs) are required to allow users to report concerns directly through: a) 24-h study helpline in the device’s help section. b) Clinical support email in the device’s help section. c) Fortnightly (or weekly for those with elevated susceptibility) interview phone calls with the research team during clinical investigations. | 2 | 2 | 4 |
| **23** | The audio that accompanies some scenarios of the intervention | 3 | 1 | 3 | 1. Within clinical investigations participants will be informed that some of the scenarios in the sessions will have audio accompanying them.  2. Study handset instructions include information about how to control the sound output | 1 | 1 | 1 |
| **24** | Content style not enjoyed by the subject | 4 | 2 | 8 | 1.The product requires ongoing clinical oversight and the ability to recall it if needed.  2.The product should detect mood changes across sessions and alert the clinical prescriber for follow-up.  3.Mood monitoring before and after each session in clinical investigations.  4.Users can report concerns via the study helpline, clinical support email, or interview calls.  5.Regular semi-structured interview calls with users to inquire about adverse events during investigations. | 1 | 1 | 1 |
| **25** | User finds the content in the sessions patronising and/or irritating | 3 | 2 | 6 | 1.The product requires ongoing clinical oversight and the ability to recall it if needed.  2.The product should detect mood changes across sessions and alert the clinical prescriber for follow-up.  3.Mood monitoring before and after each session in clinical investigations.  4.Users can report concerns via the study helpline, clinical support email, or interview calls.  5.Regular semi-structured interview calls with users to inquire about adverse events during investigations. | 1 | 1 | 1 |
| **26** | User stops or reduces existing pharmacological treatment due to taking STOP, without seeking clinical advice first   Reduced adherence to existing clinical treatments. | 5 | 4 | 20 | 1.The product must facilitate ongoing clinical treatment and oversight, with a feature allowing for clinical investigation and the ability to recall the product if necessary.  2.The product must enable users to report concerns directly via the study helpline, clinical support email, or regular interview phone calls with the research team.  3.During the clinical investigation, researchers are required to conduct semi-structured interview phone calls with users every fortnight (weekly for more susceptible individuals) to inquire about adverse events.  4.The STOP device’s labelling should emphasise that it is not intended as standalone therapy, and it does not replace a patient’s physician or ongoing treatments, including medications.  5. Requirements that for clinical investigations, researchers conduct fortnightly semi-structured interview phone calls with users which will capture nonadherence to prescribed treatment through the adverse events checklist. | 1 | 2 | 2 |
| **27** | User stops or reduces existing face to face clinical care/ treatment'   Reduced adherence to existing clinical treatment. | 5 | 4 | 20 | 1.The device must include ongoing clinical oversight and recall capability by clinical investigators if treatment discontinues. Additionally, subjects should be questioned regularly about changes in treatment as part of scheduled interactions.  2.The product should allow users to report concerns via a study helpline, clinical support email, or scheduled interviews with the research team during clinical investigations.  3.Researchers are required to conduct regular semi-structured interviews with users during clinical investigations, focusing on adverse events.  4.The labelling of the STOP device should clarify that it is not a standalone therapy and does not replace physician care or ongoing treatment.  5.During clinical investigations, researchers should conduct fortnightly interviews with users to identify non-adherence through adverse event checks.  6.Subjects should be asked about changes to treatment on a regular basis during interactions with researchers to detect inappropriate changes early. | 2 | 3 | 6 |

**Appendix G**

**Adverse Events Checklist**

| **Adverse Event Categorisation** | **Question** | **Examples/Prompts** | **Y/N** | **Adverse event description**  (e.g., details of what happened; sequence of events leading to the AE; reasons AE arose; impact on individual) | **Is the event Serious?**  0. Not serious  1. Yes, results in death  2. Yes, is life-threatening (i.e., at-risk of death)  3. Yes, requires hospitalisation or prolongation of existing hospitalisation  4. Yes, results in persistent or significant disability/incapacity  5. Yes, may jeopardise the patient, or may require intervention to prevent one of the outcomes listed above | **Severity**  1. Mild  2. Moderate 3. Severe | **Start date**  (dd/mm/yyyy) | **End date**  (dd/mm/yyyy)    (if AE is ongoing, write Not applicable) | **AE Status**  1. Ongoing - not at study end  2. Ongoing - at end of study  3. Not ongoing - recovered  4. Not ongoing - recovered with sequelae 5. Not ongoing – death | **Action taken**  1. Was action needed? (Y/N)  2. If YES, please write the code numbers for each specific subcategory of action taken as per the 'AE Actions' tab, separated by a semicolon. (For example,  ‘1.1; 1.3; 2.1; 2.2’) | **Is the AE related to the study procedure, intervention or medical device?**  1. Not related  2. Unlikely  3. Possible  4. Probable  5. Definitely | **Notes** |
| --- | --- | --- | --- | --- | --- | --- | --- | --- | --- | --- | --- | --- |
|  | **Instruction: If participant says ‘Yes’ to a question, gather further information by completing ALL remaining COLUMNS before moving to next question.** |  |  |  | **Prompt question: Were you admitted to hospital?** | **Prompt question: How much has this affected you?** | **Prompt question: When did this start?** | **Prompt question: When did it end?** |  |  | **Prompt question: Before this happened, when did you last use the app? Do you think this event was related to you using the app?** |  |
| **First, I'm going to ask you about things that are directly related to using the STOP app. I only need to know about things that have happened since we last spoke.** | | |  |  |  |  |  |  |  |  |  |  |
| Device deficiency | Have you experienced any technical difficulties with the STOP app? | Device deficiency: any technical malfunction related **ONLY** to the app itself  (e.g., STOP device ‘hangs’; App doesn’t function correctly). How often? |  |  |  |  |  |  |  |  |  |  |
| Technical malfunction | Have you experienced any other technical difficulties? | Example 1  Any technical malfunction **NOT** related to the device (i.e., app) **OR** study procedures (e.g., their TV breaks)   Example 2  Technical malfunction **NOT** related to the device (i.e., STOP app) but **IS** related to study procedures (e.g., handset, internet, HM, Qualtrics) |  |  |  |  |  |  |  |  |  |  |
| Lack of adherence to treatment | Have you had any problems with the phone or the PIN? | Forgot PIN   Lost their phone |  |  |  |  |  |  |  |  |  |  |
| Lack of adherence to treatment | **If they missed a session, ask:** Have you missed any sessions or felt like it was too much trouble? | Missed sessions or stopped using App   Does not see the benefit from using the app |  |  |  |  |  |  |  |  |  |  |
| Data breach/privacy | Have you had any concerns about privacy or security of the app?   If YES, follow up to find out if any data/ access breach **has occurred.** | Gave App access to others   Personal details from the App have been accessed by others |  |  |  |  |  |  |  |  |  |  |
| Practical burden | Has using the App caused you any inconvenience at all? Have you or anyone else noticed any negative effects of using the app? | App use leads to high phone or internet bills    Using other harmful websites on phone    Reduced time in activities of daily living |  |  |  |  |  |  |  |  |  |  |
| Content/format | What about the stories, pictures and sounds in the App? Have you had any negative reaction to those? | Startled/irritated by sound in session   Dislike content/graphics leading to disengagement |  |  |  |  |  |  |  |  |  |  |
| Assessment Related | What about the assessments (questions and jumbled word quiz) that come after the stories. How have you found those? | Increased distress or paranoia due to assessment scales   Feeling anxious thinking that they might be in control arm |  |  |  |  |  |  |  |  |  |  |
| **Now I’d like to ask you about any physical or psychological symptoms you might have been experiencing and, if you have, whether you think these are to do with the app.** | | |  |  |  |  |  |  |  |  |  |  |
| Somatic/  physical effects | Have you had any physical symptoms like tiredness, headache, eye strain, or struggling to sleep? | Eyestrain  Fatigue  Sleep disturbances  Headache |  |  |  |  |  |  |  |  |  |  |
| Symptom exacerbation/  deterioration | Have any of your mental health symptoms worsened since we last spoke? | Training items are triggering    Increased distress or paranoia changes to activities of daily living |  |  |  |  |  |  |  |  |  |  |
| Novel symptoms | Have you experienced any new mental health symptoms since we last spoke? | Trigger novel symptoms like panic attack   STOP has not reduced paranoia, leading to the user feeling demoralised or sad |  |  |  |  |  |  |  |  |  |  |
| **I'm now going to ask you about any health care you've received since we last spoke.** | | |  |  |  |  |  |  |  |  |  |  |
| Clinical care | Has there been new treatment(s) (medication, therapy…) or change in TAU? | Stops taking prescribed medication   Stops visiting physician or therapist |  |  |  |  |  |  |  |  |  |  |
| Clinical care | Have you had any emergency or unexpected contact with any health services? | A&E visits   Any hospitalisations   Unexpected or unscheduled contact with clinical services (e.g., GP visits, any hospital admissions, contact with keyworker etc.) |  |  |  |  |  |  |  |  |  |  |
| **In any clinical trial it's important to assess risk, including suicide risk. We don't expect taking part in this study will put you at risk in any way, but we still need to ask a couple of questions about your safety, is this, okay?** | | |  |  |  |  |  |  |  |  |  |  |
| Harmful behaviours | Since we last spoke, have you noticed yourself being less careful than is usual for you? For example, have you done anything you wouldn’t normally do that could be risky? | Overly trusting or uncharacteristically risky behaviours   Negatively impacted personal relationships   Using App while driving |  |  |  |  |  |  |  |  |  |  |
| Harmful behaviours | Since we last spoke, have you had any thoughts of hurting yourself or others in some way?   Since we last spoke, have you wished you were dead?   If YES, refer to the **‘Assessing suicide and self-harm risk’** flowchart. | Increased suicidal ideation   Increased self-harm   Expressed intent to harm someone else   NOTE: actual suicide attempt would be entered under 9, since would result in unplanned clinical care) |  |  |  |  |  |  |  |  |  |  |
| Other (please specify) | Thank you for your answers. I know we've covered a lot of topics today but before we finish, have you experienced anything else negative that you'd like to tell me that we have not already covered? |  |  |  |  |  |  |  |  |  |  |  |
